# A regulatory variant at 19p13.3 is associated with primary biliary cholangitis risk and ARID3A expression

**DOI:** 10.1038/s41467-023-37213-5

**Published:** 2023-03-28

**Authors:** You Li, Zhiqiang Li, Ruiling Chen, Min Lian, Hanxiao Wang, Yiran Wei, Zhengrui You, Jun Zhang, Bo Li, Yikang Li, Bingyuan Huang, Yong Chen, Qiaoyan Liu, Zhuwan Lyu, Xueying Liang, Qi Miao, Xiao Xiao, Qixia Wang, Jingyuan Fang, YongYong Shi, Xiangdong Liu, Michael F. Seldin, M. Eric Gershwin, Ruqi Tang, Xiong Ma

**Affiliations:** 1 Division of Gastroenterology and Hepatology, Key Laboratory of Gastroenterology and Hepatology, Ministry of Health, NHC Key Laboratory of Digestive Diseases, State Key Laboratory for Oncogenes and Related Genes, Renji Hospital, School of Medicine, Shanghai Jiao Tong University; Shanghai Institute of Digestive Disease, 145 Middle Shandong Road, Shanghai, China; 2 Bio-X Institutes, Key Laboratory for the Genetics of Developmental and Neuropsychiatric Disorders (Ministry of Education), Collaborative Innovation Center for Brain Science, Shanghai Jiao Tong University, Shanghai, China; 3 Affiliated Hospital of Qingdao University and Biomedical Sciences Institute of Qingdao University (Qingdao Branch of SJTU Bio-X Institutes), Qingdao University, Qingdao, China; 4 Key Laboratory of Developmental Genes and Human Diseases, Institute of Life Sciences, Southeast University, 2 Sipailou Road, Nanjing, Jiangsu China; 5 Division of Rheumatology, Department of Medicine, Allergy and Clinical Immunology, University of California at Davis, Davis, CA USA; 6 Department of Biochemistry and Molecular Medicine, University of California at Davis, Davis, CA USA; 7 Institute of Aging & Tissue Regeneration, Renji Hospital, School of Medicine, Shanghai Jiao Tong University, Shanghai, China

**Keywords:** Primary biliary cirrhosis, Gene regulation, Genome-wide association studies

## Abstract

Genome-wide association studies have identified 19p13.3 locus associated with primary biliary cholangitis (PBC). Here we aim to identify causative variant(s) and initiate efforts to define the mechanism by which the 19p13.3 locus variant(s) contributes to the pathogenesis of PBC. A genome-wide meta-analysis of 1931 PBC subjects and 7852 controls in two Han Chinese cohorts confirms the strong association between 19p13.3 locus and PBC. By integrating functional annotations, luciferase reporter assay and allele-specific chromatin immunoprecipitation, we prioritize rs2238574, an AT-Rich Interaction Domain 3A (ARID3A) intronic variant, as a potential causal variant at 19p13.3 locus. The risk allele of rs2238574 shows higher binding affinity of transcription factors, leading to an increased enhancer activity in myeloid cells. Genome-editing demonstrates the regulatory effect of rs2238574 on *ARID3A* expression through allele-specific enhancer activity. Furthermore, knock-down of *ARID3A* inhibits myeloid differentiation and activation pathway, and overexpression of the gene has the opposite effect. Finally, we find *ARID3A* expression and rs2238574 genotypes linked to disease severity in PBC. Our work provides several lines of evidence that a non-coding variant regulates *ARID3A* expression, presenting a mechanistic basis for association of 19p13.3 locus with the susceptibility to PBC.

## Abstract (web summary)

Primary biliary cholangitis is a rare, chronic immune-mediated liver disease triggered by environmental exposures in genetically susceptible individuals. Here, the authors investigate the functional mechanism underlying the association of 19p13.3 variants with primary biliary cholangitis.

## Introduction

Primary biliary cholangitis (PBC, formerly known as primary biliary cirrhosis) is the most common autoimmune liver disease, characterized by the presence of serum antimitochondrial antibodies and chronic immune-driven injury to the small and medium-sized intrahepatic bile ducts^1^. Globally, it is estimated that at least one in 1,000 women over the age of 40 years live with PBC and the risks of disease progression to cirrhosis and liver failure^2^. It is believed that PBC is triggered by genetically susceptible individuals following exposure to environmental factors^3^. Loss of tolerance occurs and results in the destruction of small bile ducts by both innate and adaptive immunity. Ursodeoxycholic acid (UDCA) is the established first-line therapeutic agent for PBC. Obeticholic acid (OCA) is emerging as a promising second-line agent for treating patients with PBC who are refractory to UDCA^4,5^. However, some patients have an inadequate response to both agents and progress to end-stage liver disease. Therefore, there is still a pressing need for additional therapeutic options based on an understanding of the pathogenesis of PBC.

Several large-scale genome-wide association studies (GWAS) in European, Japanese, and Han Chinese PBC cohorts have pointed to a strong genetic predisposition to PBC. In addition to the human leukocyte antigen (HLA) locus, more than 60 non-HLA susceptibility regions have been identified in PBC pathogenesis, including tumor necrosis factor superfamily member 15 (*TNFSF15*), Interleukin 12 A (*IL12A*), Interleukin 12 receptor (*IL12R*), Nuclear Factor Kappa B Subunit 1 (*NFKB1*) and other immune-associated genes^6–11^. For the vast majority of these susceptibility regions, the GWAS variants are present in non-coding DNA sequences and the molecular mechanisms underlying these single nucleotide polymorphisms (SNPs) remain poorly defined^12^. We recently performed a large-scale GWAS in a Han Chinese PBC cohort and identified six novel variants associated with PBC, in which we first reported that the 19p13.3 locus was associated with PBC^13^. Interestingly, this region has not been reported to be associated with any other autoimmune diseases.

To translate the results of GWAS into mechanistic insights, here we use genetic, epigenomic, high-throughput screening, and gene-editing approaches to identify the likely causal SNP at the 19p13.3 locus. Moreover, this study implicates AT-Rich Interaction Domain 3A (ARID3A) gene and its role in myeloid cells in the pathophysiology of PBC.

## Results

### Meta-analysis further supports the association of 19p13.3 region SNPs with PBC

**Fig. 1:**
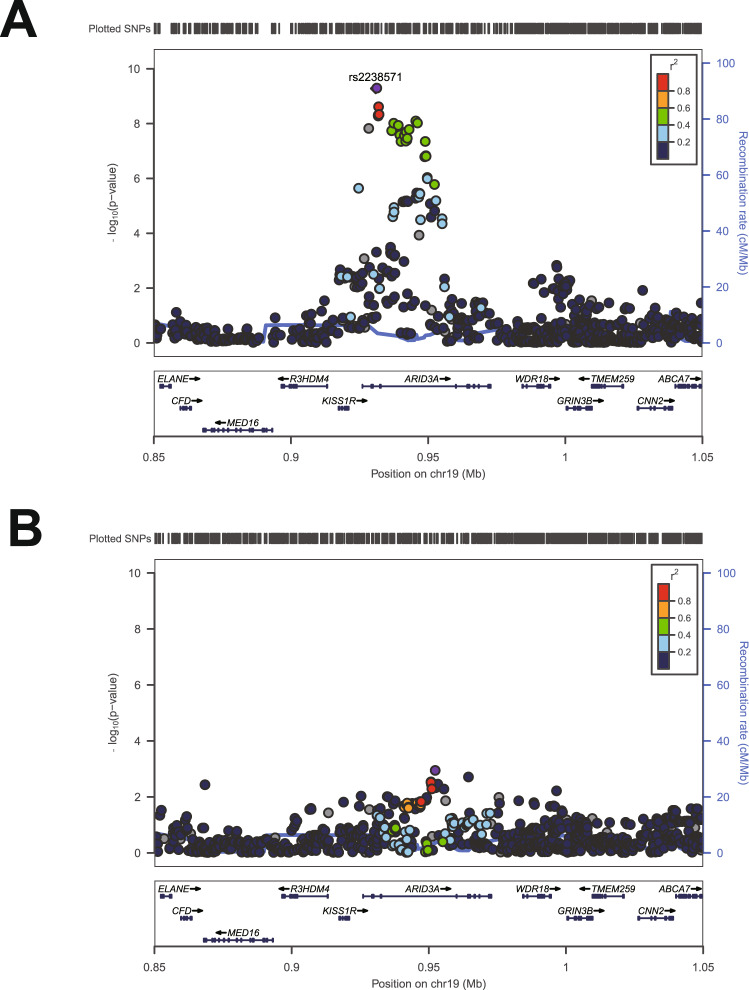
Meta-analysis of PBC association at 19p13.3. **A** Regional association plot for 19p13.3 centering on the independent SNP, rs2238571 (purple diamond). **B** Regional association plot showing the association between genetic variants and PBC at 19p13.3 after conditioning on the lead SNP.

In our previous study, 19p13.3 was first found to be associated with increased risk for PBC^13^. To further support this finding, we recruited a second Han Chinese cohort and conducted a meta-analysis that included a total of 1931 PBC subjects and 7852 controls in these two Han Chinese PBC cohorts. The results showed a strong association between 19p13.3 and PBC (rs2238571: odds ratio (OR) = 0.77, *p* = 5.24 × 10^−10^) (Fig. 1A). In addition, conditional analyses indicated that no secondary association signal was present at this locus (Fig. 1B).

To further extend these data and identify the potential causative variants for subsequent functional studies, we conducted SNP imputation at 19p13.3 from the meta-analysis results. Imputation analysis showed 20 variants that were strongly associated with PBC (*p* < 5 × 10^−8^) at 19p13.3 (Supplementary Table 1).

### In silico functional annotation implicates that the risk locus at 19p13.3 is a regulatory region and targets ARID3A

**Fig. 2:**
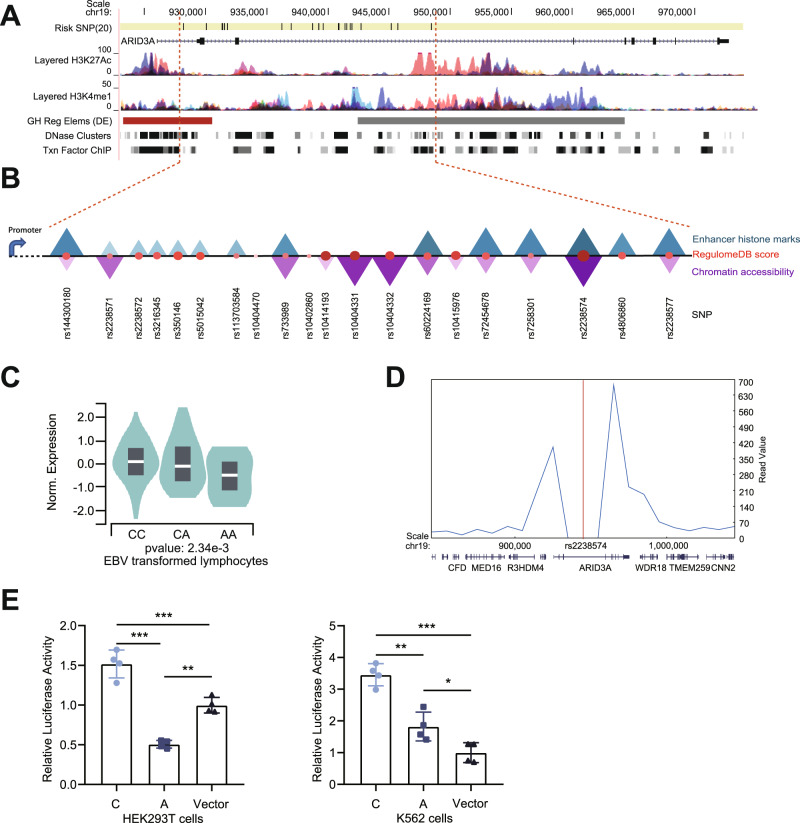
Rs2238574 is a putative regulator of *ARID3A* expression. **A** UCSC Genome Browser views of chromatin state of 19p13.3. **B** Annotation of 20 candidate variants, the size and color of the triangle and/or circle is proportional to annotation data. **C** Association between rs2238574 genotypes and expression of *ARID3A* in EBV-transformed lymphocytes (CC, *n* = 30; CA, *n* = 80; AA, *n* = 37). Source data are derived from GTEx. **D** Three-dimensional chromatin interactions for the *ARID3A* locus in K562 cells. Source data are derived from 3D Genome Browser. **E** Luciferase reporter assay in HEK293T and K562 cells transfected with pGL3 plasmids containing rs2238574. Luciferase signals are normalized to Renilla signals. Data are representative of three independent experiments (Mean ± SD for quadruplicates). *P* values are calculated using unpaired two-tailed Student’s *t* test and indicated significant if *p < 0.05, **p < 0.01, or ***p < 0.001.

The 20 significant variants reside in the intron of the *ARID3A* gene (Fig. 2A). To dissect the function of this region, we employed multiple bioinformatic tools, including the Haploreg database, rSNPBase, RegulomeDB, and ENCODE database (Fig. 2B)^14–17^. Notably, these data suggested the presence of regulatory elements at the identified loci, and therefore the risk variant(s) might confer disease susceptibility by modulating the expression of target genes. Furthermore, 7 out of the 20 variants were selected as candidate regulatory variants based on enhancer histone marks, chromatin accessibility and RegulomeDB score.

Next, we performed eQTL analysis of the 7 selected variants in EBV transformed lymphocytes from GTEx database (Fig. 2C and Supplementary Fig. 1A). The risk alleles of these variants were significantly associated with increased gene expression of *ARID3A*. To further verify the putative target gene of this regulatory region, we measured the three-dimensional chromatin topology of this region using the published Hi-C dataset, and observed a physical interaction between the risk locus and *ARID3A* promoter (Fig. 2D and Supplementary Fig. 1B). It was essential to determine the cell type before investigating the function of the variants. We explored the expression pattern of *ARID3A* in a range of immune cells. *ARID3A* was highly expressed in myeloid cells and markedly up-regulated in differentiated myeloid cells^18,19^, suggesting that ARID3A might play a role in myeloid cells (Supplementary Fig. 1C, D).

### Evidence that rs2238574 determines enhancer activity

Given that the region showed evidence of regulatory function, we tested whether the risk alleles affect gene transcription efficiency using an enhancer luciferase reporter assay. Among the 7 tested variants, rs2238574 and rs2238577 showed differential regulatory activity between the two alleles in both HEK293T cells and K562 cells (Fig. 2E and Supplementary Fig. 2). Specifically, the region with the risk allele showed significantly higher enhancer efficiency than the region with the non-risk allele, consistent with the eQTL results aforementioned. Considering functional annotations and luciferase reporter results, we chose rs2238574 as the candidate SNP for follow-up experiment.

### Rs2238574 affects transcription factor binding

**Fig. 3:**
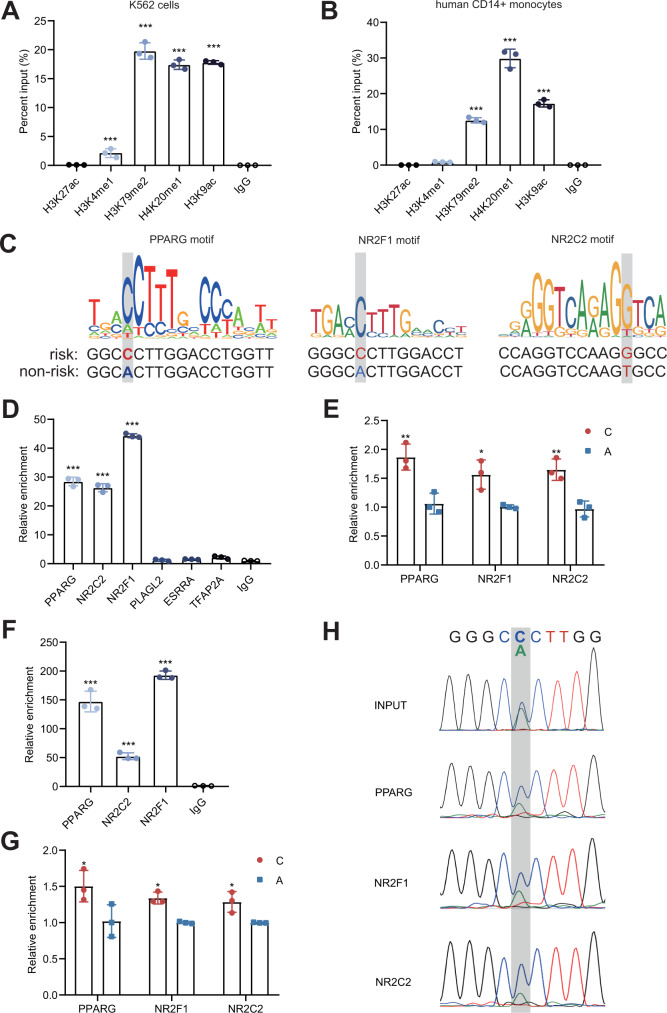
The risk allele of rs2238574 enhances the chromatin binding of transcription factors. **A**, **B** ChIP-qPCR confirmation of the enrichment of active enhancer marks at rs2238574-containing region in K562 cells and human CD14^+^ monocytes. **C** Motif analysis suggests that rs2238574 resides within DNA-binding motifs of PPARG, NR2F1 and NR2C2. **D** ChIP-qPCR results showing enrichment of PPARG, NR2F1 and NR2C2 at the rs2238574-containing region in K562 cells. **E** ChIP-AS-qPCR showing allele-specific binding of PPARG, NR2F1 and NR2C2 at rs2238574 in K562 cells. **F** ChIP-qPCR results showing enrichment of PPARG, NR2C2 and NR2F1 at the rs2238574-containing region in human CD14^+^ monocytes. **G** ChIP-AS-qPCR showing allele-specific binding of PPARG, NR2C2 and NR2F1 at rs2238574 in human CD14^+^ monocytes. **H** Sanger sequencing chromatograms of the SNP region from the input and ChIPed DNA in human CD14^+^ monocytes. Data are representative of three independent experiments (Mean ± SD for triplicates). *P* values are calculated using unpaired two-tailed Student’s *t* test and indicated significant if *p < 0.05, **p < 0.01, or ***p < 0.001.

To better understand the regulatory function of rs2238574, we examined the chromatin state maps of this region in different immune cell types using publicly available epigenetic data^14^. We observed an enrichment of epigenetic marks for active enhancers at rs2238574-containing region in human CD14^+^ monocytes (Supplementary Fig. 3A). In contrast, there were no detectable signals of those markers in human CD19^+^ B cells, CD4^+^ T cells and CD8^+^ T cells. Thus, we proposed that the rs2238574-harboring region may be a cell-specific enhancer for myeloid cells. This observation was supported by ChIP-seq data in human myeloid cell lines, K562 cells. Additional chromatin immunoprecipitation followed by qPCR (ChIP-qPCR) in K562 cells and primary human CD14^+^ monocytes further validated the enrichment of histone markers H3K4me1, H3K79me2, H3K20me1 and H3K9ac at this region (Fig. 3A, B).

Regulatory SNPs with causal roles in disease susceptibility often affect gene expression by modulating transcription factor binding. Bioinformatic analysis using JASPAR, TRANSFAC and CIS-BP database suggested multiple transcription factors binding to the rs2238574-containing region. Among them, many transcription factors, such as  peroxisome proliferator activated receptor gamma (PPARG), nuclear receptor subfamily 2 group F member 1 (NR2F1), nuclear receptor subfamily 2 group C member 2 (NR2C2), transcription factor AP-2 alpha (TFAP2A), pleiomorphic adenoma gene-like 2 (PLAGL2) and estrogen related receptor alpha (ESRRA) were predicted to have a higher preference for the risk allele (C) (Fig. 3C and Supplementary Fig. 3B). The ChIP-qPCR results validated the enrichment of PPARG, NR2F1 and NR2C2 at this region in K562 cells (Fig. 3D). However, TFAP2A, PLAGL2 and ESRRA indicated no binding activity to this region in vitro.

To further evaluate the binding preference of these transcription factors to rs2238574, we conducted ChIP followed by allele-specific qPCR (AS-qPCR) analysis in K562 cells that were heterozygous at this SNP (Fig. 3E). Strikingly, the risk-associated C allele of rs2238574 exhibited a higher binding affinity for PPARG, NR2F1 and NR2C2 relative to that of the non-risk allele.

Then, we performed ChIP-qPCR in primary human CD14^+^ monocytes that are heterozygous for rs2238574. Consistently, the results showed a strong occupancy of PPARG, NR2F1 and NR2C2 at the DNA fragment harboring rs2238574 and binding preference to the risk allele (Fig. 3F, G). In line with this finding, Sanger sequencing analysis showed that the risk-associated C allele was enriched in chromatin fragments immunoprecipitated with antibodies to PPARG, NR2F1 and NR2C2 (Fig. 3H). In addition, we found that *ARID3A* expression was positively correlated with the expression of *PPARG* or *NR2C2* in whole blood from GTEx dataset (Supplementary Fig. 3C). Our data thus strongly suggest that the risk allele of rs2238574 up-regulates expression of *ARID3A* by altering DNA-binding affinity of transcription factors.

### ARID3A modulates myeloid cell differentiation and activation

**Fig. 4:**
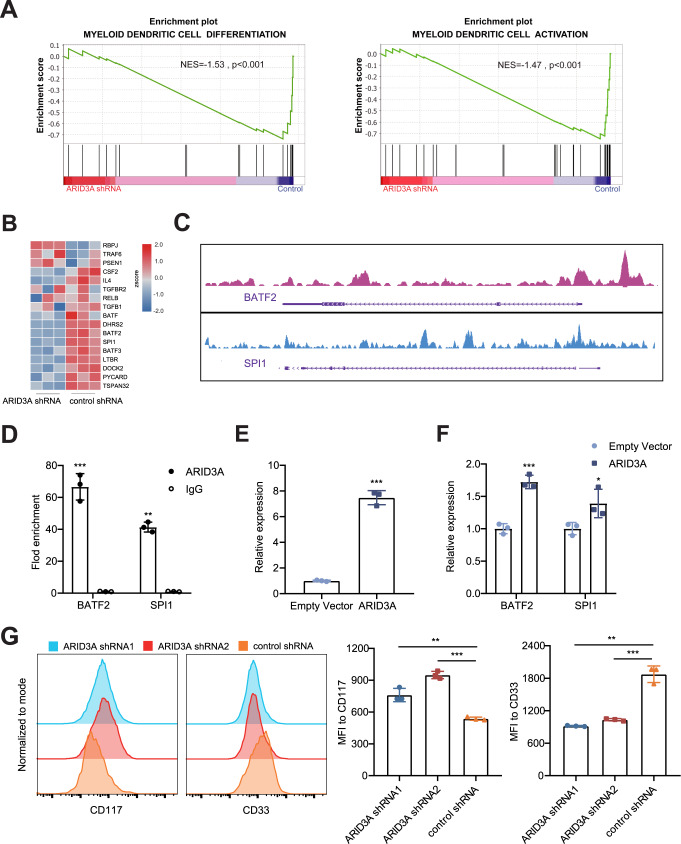
ARID3A contributes to the differentiation of myeloid cells. **A** GSEA showing the myeloid differentiation pathway and myeloid activation pathway were downregulated in *ARID3A* knock-down K562 cells. **B** Heatmap of differentially expressed genes related to myeloid differentiation pathway and myeloid activation pathway in *ARID3A* knock-down K562 cells (three replicates). **C** ARID3A ChIP-seq signals at the promoter region of *BATF2* and *SPI1* in K562 cells. Source data are derived from ENCODE. **D** ChIP-qPCR confirmation of the enrichment of ARID3A at the promoter region of *BATF2* and *SPI1* in K562 cells. **E** Increased expression of *ARID3A* after *ARID3A* plasmid overexpression in K562 cells. **F** Relative expression of *BATF2* and *SPI1* after *ARID3A* plasmid overexpression in K562 cells. **G** Flow cytometric measurement of surface antigen CD117 and CD33 after knock-down of *ARID3A* in K562 cells. Data are representative of three independent experiments (Mean ± SD for triplicates). *P* values are calculated using unpaired two-tailed Student’s *t* test and indicated significant if *p < 0.05, **p < 0.01, or ***p < 0.001. MFI mean fluorescence intensity.

To explore the role of ARID3A in myeloid cells, we conducted lentivirus-mediated short hairpin RNA (shRNA) against *ARID3A* in K562 cell line, followed by RNA sequencing (RNA-seq). Gene set enrichment analysis (GSEA) suggested that the myeloid differentiation pathway and myeloid activation pathway were downregulated in *ARID3A* knock-down cells (Fig. 4A, B). Real-time qPCR confirmed that *ARID3A* knock-down greatly decreased expressions of *BATF*, *BATF2*, *BATF3* and *SPI1* (Supplementary Fig. 4A, B). Consistently, we observed an enrichment of transcription factor ARID3A at the promoter region of *BATF2* and *SPI1* in K562 cells based on ChIP-seq profiles from ENCODE (Fig. 4C). Using ChIP-qPCR, we next validated that ARID3A could directly bind to the promoter region of *BATF2* and *SPI1* gene in K562 cells and thus up-regulate their expression (Fig. 4D). As expected, overexpression of *ARID3A* in K562 cells significantly increased expression of *BATF2* and *SPI1* (Fig. 4E, F). In support of our findings, we found that *ARID3A* expression was positively correlated with the expression of *BATF2* and *SPI1* in whole blood from the GTEx dataset (Supplementary Fig. 4C).

To clearly define whether ARID3A was involved in myeloid differentiation, we performed flow cytometry analysis and found that compared with control counterparts, knock-down of *ARID3A* significantly led to decreased expression of *CD33* (a myeloid marker) and increased expression of *CD117* (a well-established stem cell marker) in K562 cells (Fig. 4G). Conversely, overexpression of *ARID3A* led to robust decreased expression of *CD117*, whereas no significant effects on expression of *CD33* were observed (Supplementary Fig. 4D). To further investigate the role of ARID3A in primary myeloid cell, we used shRNA to effectively knock-down *ARID3A* in monocyte-derived macrophages (MDMs). The flow cytometry analysis showed that *ARID3A* knock-down significantly decreased expression of *CD68* (a macrophage marker), further supporting the effect of ARID3A on myeloid differentiation (Supplementary Fig. 5A). In addition, knock-down of *ARID3A* significantly led to decreased expression of pro-inflammatory cytokines, including *IL1B* and *IL8* (Supplementary Fig. 5B). As for pro-fibrotic cytokines, expression of *PDGFB* was reduced in *ARID3A* knock-down MDMs (Supplementary Fig. 5C). Taken together, ARID3A may play an important role in myeloid differentiation and function.

### Genome editing provides additional evidence that rs2238574 regulates *ARID3A* expression and myeloid cell differentiation

**Fig. 5:**
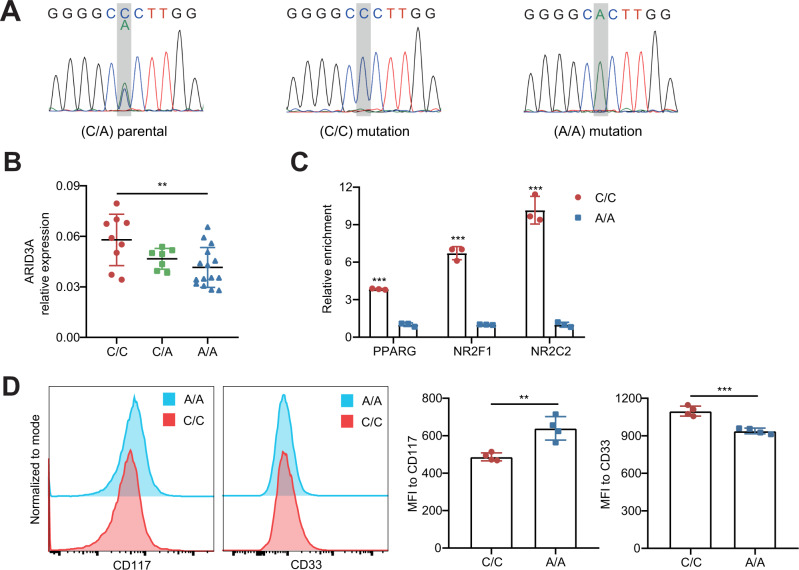
Effects of rs2238574 genotypes on *ARID3A* expression in K562 cells. **A** Sanger sequencing of CRISPR/Cas9-modified and parental K562 cells. **B** Relative expression of *ARID3A* in mutated and parental K562 cells (C/C, *n* = 9; C/A, *n* = 7; A/A, *n* = 15). **C** ChIP-qPCR results showing different enrichment of PPARG, NR2F1 and NR2C2 at the rs2238574-containing region between C/C and A/A K562 cells (*n*  =  3, biologically independent samples). **D** Flow cytometric measurement of surface antigen CD117 and CD33 in C/C and A/A K562 cells. Data are representative of three independent experiments (Mean ± SD for quadruplicates). *P* values are calculated using unpaired two-tailed Student’s *t* test and indicated significant if *p < 0.05, **p < 0.01, or ***p < 0.001. MFI mean fluorescence intensity.

We applied CRISPR/Cas9-mediated genome editing to generate K562 cells with different genotypes (Fig. 5A). Compared with the cells with A/A genotype, we observed an increased *ARID3A* expression in the cells with C/C genotype (Fig. 5B). In addition, we observed higher levels of transcription factors chromatin occupancy at rs2238574-containing region for PPARG, NR2F1 and NR2C2 in the mutated cells with C/C than that in mutated cells with A/A (Fig. 5C). We also analyzed the phenotypic difference between mutated cells and found that the C/C genotype K562 cells had a lower expression of *CD117* and a higher expression of *CD33* than A/A genotype cells (Fig. 5D). Collectively, these results demonstrate that the risk allele of rs2238574 upregulates the expression of *ARID3A* and affects the differentiation of K562 cells.

### Increased *ARID3A* expression in PBC

**Fig. 6:**
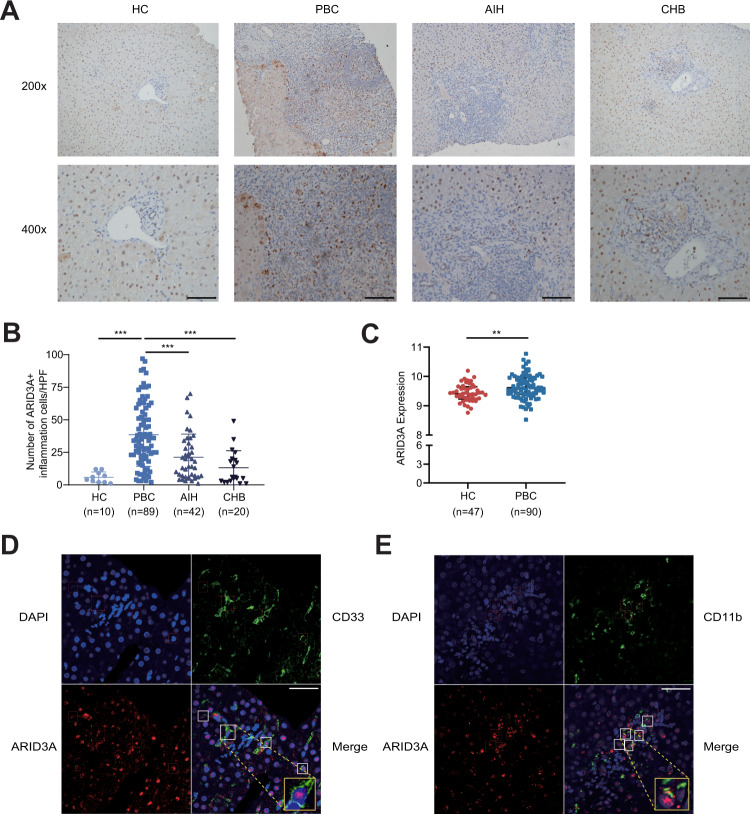
Increased *ARID3A* expression in PBC. **A**, **B** Representative immunohistochemical staining (x200; x400) and statistical analysis of ARID3A in HC (*n* = 10), PBC (*n* = 89), AIH (*n* = 42) and CHB (*n* = 20) patients. **C** Increased *ARID3A* expression in whole blood from PBC patients (HC, *n* = 47; PBC, *n* = 90). **D** Representative confocal staining of CD33 (in green), ARID3A (in red) and DAPI (for nuclei in blue) in the PBC liver (*n* = 10). **E** Representative confocal staining of CD11b (in green), ARID3A (in red) and DAPI (for nuclei in blue) in the PBC liver (*n* = 10). Data are represented as mean ± SD. *P* values are calculated using unpaired two-tailed Student’s *t* test and indicated significant if *p < 0.05, **p < 0.01, or ***p < 0.001. Scale bars, 20 μm. HC healthy control, AIH autoimmune hepatitis, CHB chronic hepatitis B.

To determine whether *ARID3A* is expressed differentially in PBC, we quantified its expression in liver samples from patients of different liver diseases and healthy controls (HCs). IHC staining of the diagnostic liver biopsy showed that the expression of *ARID3A* in the portal area of PBC was significantly increased, compared with autoimmune hepatitis (AIH), chronic hepatitis B (CHB) and HCs (Fig. 6A, B). We next explored *ARID3A* expression in whole blood using a transcriptome dataset from 90 PBC and 47 HCs^20^. The results showed a marginal but significant increase in *ARID3A* expression in whole blood cells from PBC compared to HCs (Fig. 6C). Furthermore, confocal staining demonstrated that ARID3A colocalized with myeloid markers CD33 and CD11b in PBC liver (Fig. 6D, E).

### *ARID3A* expression and rs2238574 genotypes are associated with disease severity

**Fig. 7:**
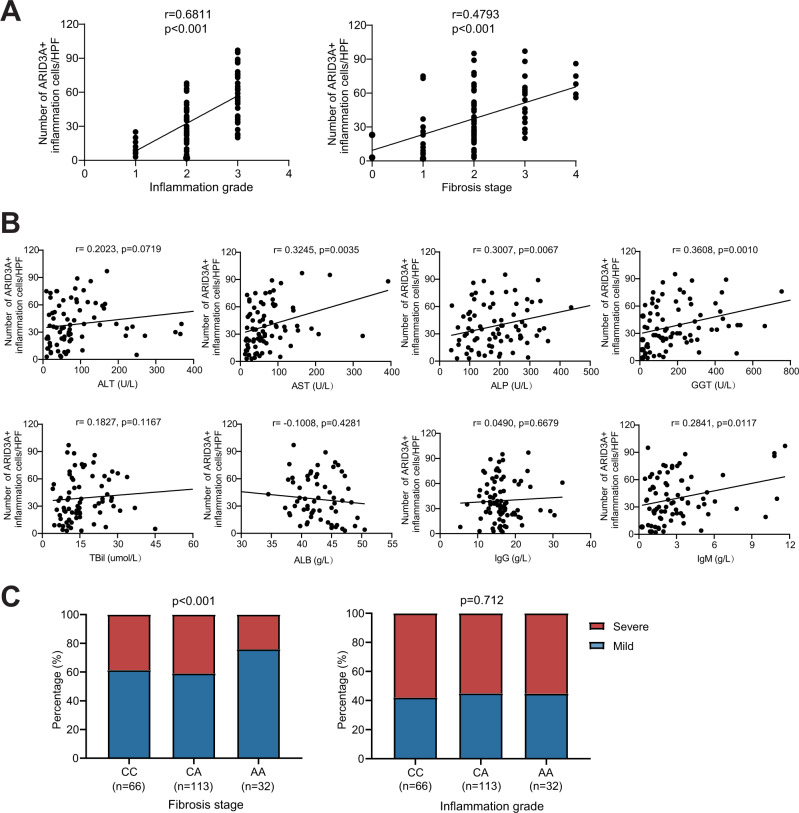
*ARID3A* expression and rs2238574 genotypes are associated with disease severity. **A** Association of the numbers of hepatic ARID3A positive inflammation cells with hepatic inflammatory degrees (left) and fibrosis stages (right) in PBC patients. **B** Association of the numbers of hepatic ARID3A positive inflammation cells with serum ALT, AST, GGT, ALP, TBil, ALB, IgG and IgM levels. Association analysis are performed using Pearson’s correlation. **C** Association of rs2238574 genotypes with liver fibrosis (left) and inflammation (right) in subjects with PBC (C/C, *n* = 66; C/A, *n* = 113; A/A, *n* = 32). Correlation analysis are performed using logistic regression adjusted for disease history. ALT Alanine aminotransferase, AST aspartate transaminase, ALP alkaline phosphatase, GGT γ-Glutamyl transferase, TBil Total bilirubin, ALB albumin, IgG immunoglobulin G, IgM immunoglobulin M.

To evaluate the clinical significance of elevated *ARID3A* expression, we analyzed the expression of *ARID3A* based on liver histopathological features and clinical characteristics in PBC. We found that the number of ARID3A positive inflammation cells in liver tissue was positively correlated with liver inflammation grade (*r* = 0.6811, *p* < 0.001) and fibrosis stage (*r* = 0.4793, *p* < 0.001) (Fig. 7A). In addition, the number of ARID3A positive inflammation cells in PBC had a strong correlation with aspartate transaminase (AST) (*r* = 0.3245, *p* = 0.0035), alkaline phosphatase (ALP) (*r* = 0.3007, *p* = 0.0067), γ-Glutamyl transferase (GGT) (*r* = 0.3608, *p* = 0.0010) and immunoglobulin M (IgM) (*r* = 0.2841, *p* = 0.0117) (Fig. 7B).

Given that rs2238574 correlates with *ARID3A* expression, we also investigated whether rs2238574 genotypes were directly correlated with clinical features of PBC. We genotyped rs2238574 in 211 patients with diagnostic liver biopsies. The stage of fibrosis and inflammation was divided into two levels: mild and severe, according to the Scheuer scoring system^21^. We found a significant association between the C risk allele of rs2238574 and higher stage of liver fibrosis after correcting for disease history (Fig. 7C). However, there was no significant association between the genotypes of rs2238574 with liver inflammation grade.

## Discussion

GWAS have collectively identified over 60 loci contributing genetic susceptibility to PBC, the vast majority of which are located in non-coding regions of the genome. However, it has been challenging to identify the causative variants and elucidate their functions. To translate the results of GWAS into mechanistic insights, here we devised a strategy to systematically investigate the underlying mechanisms of PBC risk loci.

**Fig. 8:**
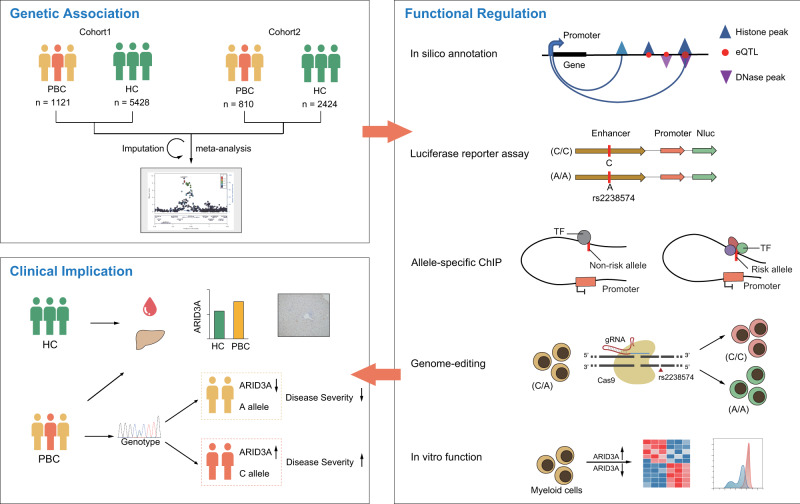
Graphical representation of the regulation and function of 19p13.3 in PBC. HC healthy control, ChIP chromatin immunoprecipitation, TF transcription factor.

In this study, we applied genetic, epigenomic and genome-editing approaches to a PBC-associated 19p13.3 locus and identified rs2238574, an *ARID3A* intronic SNP, as the putative disease causal variant. The risk genotype of rs2238574 increased the enhancer activity, leading to an elevated expression of *ARID3A* by altering the DNA-binding affinity of transcription factors in myeloid cells. At the functional level, the data supported an important role for ARID3A in the differentiation and function of myeloid cells. At the clinical level, the risk genotype and *ARID3A* expression were associated with disease severity (Fig. 8).

The majority of cis-regulatory interactions are usually highly cell-type specific^22^. Intriguingly, we observed a significant enrichment of epigenetic marks for active enhancers at rs2238574-containing region in human CD14^+^ monocytes, but not in CD19^+^ B cells, CD4^+^ T cells or CD8^+^ T cells. Thus, we proposed that the rs2238574-containing region may be a myeloid cell-specific enhancer. Recent scRNA-seq data also revealed that *ARID3A* was highly expressed in myeloid cells, especially mature myeloid cells^18,19^. Accordingly, we sought to dissect the function of the causal variant and its target gene in myeloid cells. We found that transcription factors including *PPARG*, *NR2G1* and *NR2C2*, preferentially bound the risk allele of rs2238574, which led to increased expression of *ARID3A*.

ARID3A is a member of a large family of A + T-rich interaction domain (ARID) proteins^23,24^. ARID3A was originally named Bright (B cell regulator of immunoglobulin (Ig) heavy chain transcription) for its ability to enhance the transcription of immunoglobulin heavy chains in antigen-activated B cells^25^. Recent studies further demonstrated that ARID3A was not only crucial for B cell lineage function but also participated in the development of placental and hematopoietic stem cells^26–29^. Moreover, *ARID3A* expression was upregulated in B cells, and plasmacytoid dendritic cells from patients with systemic lupus erythematosus (SLE) and correlated with disease severity^30,31^. It was reported that ARID3A could induce the production of interferon-alpha, a major inflammatory cytokine in SLE^32,33^.

Myeloid cells, i.e., monocytes and macrophages, represent a key component of innate and adaptive immune systems and play an important role in the pathogenesis of PBC. CD68^+^ monocytes/macrophages are enriched in/around injured bile ducts of PBC^34^. Additionally, monocytes/macrophages from PBC patients are hypersensitive to infectious stimuli and produce a variety of cytokines and chemokines that amplify the immune response and further aggravate the damage of cholangiocytes^35,36^. At the same time, macrophages secrete profibrotic mediators, such as transforming growth factor-beta and platelet-derived growth factor, resulting in the activation of hepatic stellate cells and liver fibrosis^36,37^. We have previously shown that myeloid-derived suppressor cells (MDSCs), a group of immature myeloid cells, were expanded in the periphery of PBC patients and exerted immunosuppressive effects on T cell proliferation^38^. The current study points to new roles of ARID3A in myeloid cells, which not only promotes differentiation of myeloid cells but also enhances cytokine secretion of macrophages.

Although GWAS have improved our understanding of disease pathogenesis, application of these data to clinical practice is challenging and efforts have been made to interpret GWAS findings^39,40^. Here, we found that the risk SNP genotype and *ARID3A* expression were associated with disease severity, providing additional value in patient stratification. Furthermore, the mechanistic study implicated ARID3A in myeloid cell differentiation and function, providing further opportunities for PBC intervention by manipulation of ARID3A or myeloid cell pathway.

There were several limitations of this study. While our study shows the effect of rs2238574 on myeloid cells in PBC, we cannot exclude the possibility that additional variants at this locus may also affect disease phenotype and present additional or alternate hypothesis as to causal effects. In addition, the functional relevance of 19p13.3 and ARID3A in the development of PBC was investigated only in a limited number of clinical samples. Further studies in larger sample sizes to more powerfully examine clinical correlations will be necessary. Lastly, genetically engineered mouse models are warranted to further explore the role of ARID3A variants in PBC.

In summary, we provide several lines of evidence to elucidate the functional mechanism underlying the association of 19p13.3 variants with PBC. The putative causal variant rs2238574 at 19p13.3 regulates *ARID3A* expression and may contribute to myeloid cell differentiation and function, which not only facilitates the understanding of PBC at a genome-wide level, but also suggests that this pathway might present an opportunity for novel therapeutic strategies in this disease.

## Methods

### Study population

We carried out a case/control association study in two independent cohorts and then performed a meta-analysis. Cohort 1 was from a previously published GWAS, which comprised 1121 PBC patients and 5467 controls^13^. In cohort 2, 820 PBC cases were recruited from Renji Hospital, Shanghai Jiao Tong University, and underwent genome-wide genotyping for the first time; 3194 controls were recruited from Shanghai Jiao Tong University and matched to cases by age and gender. All participants were genetically unrelated individuals of self-claimed Chinese Han descent.

The diagnosis of PBC was based on the criteria recommended by American Association for the Study of Liver Diseases (AASLD) and the European Association for the Study of the Liver (EASL)^41,42^. The study was conducted in accordance with Declaration of Helsinki and approved by the research ethics boards of Renji Hospital, Shanghai Jiao Tong University. Written consent forms were obtained from all the subjects.

### Genotyping and quality control

Both cases and controls in cohort 1 were genotyped with Han Chinese population-specific HumanOmniZhongHua-8 BeadChip, version 1.1 (Illumina, San Diego, CA), and cohort 2 were genotyped using Infinium Global Screening Array, version 3.0 (Illumina, San Diego, CA).

We performed systematic quality control on the raw genotyping data to filter out both unqualified samples and SNPs using the Ricopili pipeline for case-control groups^43^. Samples with low SNP call rate (<98%) as well as individuals closely related based on estimated identity-by-descent (PI_HAT > 0.25) were excluded for further analysis. Sex was established via genotyping and samples with inconsistent sex (compared with the sample record) were removed. SNPs with call rates <98%, MAF < 0.5%, or significant deviation from Hardy–Weinberg equilibrium (HWE) in cases (*p* < 1 × 10^−10^) or controls (*p* < 1 × 10^−6^) were excluded. The remaining samples were subsequently assessed for population stratification using principal component analysis (PCA), which was performed by the EIGENSTRAT software. After quality control filtering, genotype data for 776516 variants in 1931 cases and 7852 controls (cohort1 contained 1121 cases and 5428 controls; cohort 2 contained 810 cases and 2424 controls) remained.

### Imputation and association analysis

The genotypes were phased with SHAPEIT (URLs)^44,45^ for each chromosome, and imputation was performed with IMPUTE2 (URLs)^46^ and based on data from 1000 Genomes Project (phase 3)^47^. The variants with INFO > 0.8, MAF > 0.01, a call rate ≥98% and HWE (*P* ≥ 1 × 10^−6^) in the controls were included for further analysis.

Association analyses of each cohort were performed with logistic regression in the Ricopili pipeline and age, sex and ten principal components were used as covariates in the association analysis to correct for the population stratification^43^. We then carried out a meta-analysis using the inverse-variance fixed-effects method to combine the results from two cohorts’ datasets using META (version 1.761). The genome-wide significance threshold was set at *p* < 5 × 10^–8^ and SNPs that were not nominally significant in both cohorts (*p* < 0.05) were excluded.

To identify additional independent signals at 19p13.3, we carried out the conditional analysis in which we included the allele count of the lead variant (rs2238571) as a covariate in the model. The regional association results were created with LocusZoom (version 1.2)^48^. Linkage disequilibrium (LD) was estimated from the imputation reference panel.

### Cell lines

Human cell lines, human embryonic kidney 293T (HEK293T) and myeloid leukemia K562 were used in the study. All cell lines were purchased from the Cell Bank of the Chinese Academy of Sciences in Shanghai and cultured according to ATCC culture methods. HEK293T cell lines were cultured in Dulbecco’s Modified Eagle Medium supplemented with 10% fetal bovine serum (FBS). K562 cell lines were cultured in Iscove’s Modified Dulbecco’s Medium supplemented with 10% FBS. Cell lines were maintained at 37 °C in humidified CO_2_ (5%) incubators. All the cell lines were authenticated via short tandem repeat fingerprinting and tested negative for mycoplasma.

### Luciferase enhancer reporter assay

The double-stranded oligonucleotide containing the SNP of interest was cloned upstream from the luciferase gene in the luciferase reporter vector pGL3 promoter (Promega, USA). For HEK293T cells, 1 × 10^4^ HEK293T cells were transfected with 100 ng of pGL3-Promoter vector along with 10 ng of internal pRL-TK Renilla luciferase vector (Promega, USA) using Lipofectamine 3000 reagents (Thermo-Fisher Scientific, USA) according to the manufacturer’s instructions. For K562 cells, 2 × 10^5^ K562 cells were transfected with 1 µg of pGL3-Promoter vector along with 100 ng of pRL-TK Renilla luciferase vector using Neon Transfection System (Thermo-Fisher Scientific, USA). After 48 h of transfection, the HEK293T and K562 cells were collected respectively for luciferase activity measurement using a Dual-Luciferase Reporter Assay System (Promega, USA). Firefly luciferase activity was expressed as relative luciferase activity after correction for Renilla luciferase activity to adjust for transfection efficiency. At least three independent transfection experiments for each construct were performed.

### Primary human monocytes isolation and cell culture

Whole blood was collected from healthy human donors with approval by the research ethics boards of Renji Hospital, Shanghai Jiao Tong University. Peripheral blood mononuclear cells (PBMCs) were isolated by Ficoll gradient centrifugation and primary human CD14^+^ monocytes were isolated from PBMCs using CD14 MicroBeads (Miltenyi, Germany) according to the manufacturer’s instructions. Isolated monocytes were cultured with 10 ng/ml macrophage colony-stimulating factor (Peprotech, USA) in 10% FBS–containing RPMI 1640 Medium for 7 days to generate monocyte-derived macrophages (MDMs).

### DNA extraction and SNP genotyping

Genomic DNA was extracted from human cell lines and primary human cells using Blood Genomic DNA Extraction Kit (Tiangen Company, China). SNP genotyping was performed by Sanger DNA sequencing.

### Chromatin immunoprecipitation

Chromatin immunoprecipitation (ChIP) assays were performed using EZ-Magna ChIP A/G Chromatin Immunoprecipitation Kit (Sigma-Aldrich, USA) according to the manufacturer’s instructions. Briefly, 2 × 10^7^ cells were fixed with 1% formaldehyde for 10 min, and then 2 mL of 10 × Glycine was added to quench unreacted formaldehyde. After being washed twice with cold PBS, cells were then collected for nuclear extraction by cell lysis buffer and finally resuspended in 0.5 mL of nuclear lysis buffer. Nuclear lysates were subsequently sonicated using the Covaris ultrasonicator. The supernatant was immunoprecipitated with 5 μg of the antibody of interest, including PAR Gamma Polyclonal Antibody (Proteintech, #16643-1-AP), COUP TF1 Antibody (GeneTex, #GTX114835) and Anti-TR4 Antibody (Abcam, #ab109301), and 20 µL of fully resuspended protein A/G magnetic beads overnight at 4 °C with rotation. Normal rabbit IgG (CST, #2729) was used as a negative control. qPCR was performed on immunoprecipitated chromatin to determine transcription factors of interest enrichment occupancy on the rs2238574-containing region.

Allele-specific quantitative RT-PCR (AS-qPCR) was performed similarly to normal qPCR. The primers were designed for allele-specific amplification of the rs2238574 region with a C or A allele in the DNA samples from ChIP.

### Single nucleotide mutation using CRISPR/Cas9

We used prime editing to improve the efficiency of single nucleotide mutation^49^. PegRNAs and Nicking sgRNA were designed using CHOPCHOP (version 3) (http://chopchop.cbu.uib.no/)^50^.

For prime editing, 1.5 × 10^6^ K562 cells were electroporated with 7.5 μg pCMV-PE2-P2A-GFP plasmid, 2.5 μg pegRNA plasmid, and 830 ng nicking sgRNA plasmid using 100 μL Neon Transfection System (Thermo Fisher, USA)^49^. After transfection for 3 days, single cells with high GFP fluorescence were sorted into 96-well plates supplemented with 200 μL culture medium in each well for clone selection. Following 21 days of cell growth, cells were then harvested for DNA sequencing.

### Lentiviral virus infection

3 × 10^5^ cells were seeded in 12-well plates and transduced with lentiviral particles. 24 h post-infection, the virus was removed and replaced by normal medium containing final 3 mg/ml puromycin (Sigma-Aldrich, USA). When uninfected control cells completely died, the target cells were cultured in normal growth medium with 0.5 mg/ml puromycin.

### RNA extraction and quantitative RT-PCR

Total RNA was extracted and purified from K562 cells using RNAiso Reagent (Takara, Japan) according to the manufacturer’s instructions. 1 μg of total RNA was reverse transcribed using the PrimeScript RT Reagent Kit (Takara, Japan) to detect relative mRNAs. Real-time PCR was performed in triplicates on an Applied Biosystem7900 quantitative PCR system (Applied Biosystems, USA) using TB Green Premix Ex Taq reagent (Takara, Japan). The Ct values obtained from different samples were compared using the 2^−ΔCt^ method. GAPDH served as the internal reference gene.

### RNA-seq and differential expression analysis

RNA samples were prepared from *ARID3A* knock-down and control K562 cells, each with three biological replicates. RNA quality was quantified and qualified by Agilent 2100 Bioanalyzer (Agilent Technologies, USA). Total RNA (1 μg) with RNA integrity number (RIN) value above or equal to 8 was used for the following library preparation. Total Barcoded RNA-seq libraries were sequenced as 150-bp paired-end reads using the Illumina Novaseq platform. Raw data of fastq format were firstly processed through in-house Perl scripts. In this step, clean data were obtained by removing reads containing adapter, reads containing ploy-N and low-quality reads from raw data. All the downstream analyses were based on the clean data with high quality. Differential expression analysis of two groups (three biological replicates per group) was performed using the DESeq2 R package. Sample plots and differential expression were conducted using R software. Gene Set Enrichment Analysis was performed using GSEA (version 4.2.3).

### Flow cytometry analysis

Briefly, cells were harvested and washed with PBS before staining and then incubated with antibodies for 30 min in the dark at 4 °C. For intracellular staining, surface-stained cells were fixed and permeabilized with Cytofix/Cytoperm solution (BD Biosciences, USA) for 20 min at 4 °C and then were stained with antibodies for 30 min at 4 °C. The following antibodies were used: anti-CD33 (BioLegend, #366622), anti-CD117 (BioLegend, #313232) and anti-CD68 (BD Biosciences, #564943). Subsequently, cells were finally washed with PBS and then analyzed by flow cytometry (BD Biosciences, USA). A total of 500,000 events were recorded and analyzed using FlowJo software, version 10.6.2 (Tree Star, USA). The full gating strategy is shown in Supplementary Fig. 6.

### Histological staining

Formalin-fixed, paraffin-embedded liver tissues were obtained from ultra-sound-guided needle liver biopsies of 89 patients with PBC, 42 with autoimmune hepatitis (AIH), 20 with chronic hepatitis B (CHB), and 10 healthy controls (HC). For immunohistochemistry (IHC) and immunofluorescence (IF) staining, paraffin-embedded liver sections were first incubated with primary antibodies against ARID3A (LSBio, #LS-B5399), CD33 (Abcam, #ab26945), CD11b (Abcam, #ab133357). For IHC staining, the sections were then incubated with HRP-conjugated secondary antibody, followed by 3′-diaminobenzidine (DAB) for visualization in light microscopy. For IF staining, the sections were then incubated with fluorochrome-conjugated secondary antibody (Invitrogen, USA), followed by histological observation using laser confocal microscopy (Carl Zeiss, Germany). All the sections were analyzed by a hepatic pathologist, and five random fields were selected for each section. The numbers of ARID3A positive inflammatory cells were quantified at 40 × 10 magnification. Inflammatory degrees and fibrotic stages were evaluated according to the Scheuer scoring system^21^.

### Statistical analysis

Differences in continuous variables were compared by a two-tailed Student *t*-test or Mann–Whitney *U* test, where applicable. Categorical variables were assessed by chi-squared test or Fisher’s s exact test as appropriate. Correlations were performed using Pearson’s correlation. All statistical analyses were performed using statistical package SPSS 22.0 (SPSS Inc, USA) and RStudio (version 1.1.463) with R (version 3.6.3). All of the *P* values were shown as two-sided, and *P* < 0.05 was considered statistically significant.

### Reporting summary

Further information on research design is available in the Nature Portfolio Reporting Summary linked to this article.

## Supplementary information

Supplementary Information

Reporting Summary

## Data Availability

Encyclopedia of DNA Elements database (https://www.encodeproject.org/), HaploReg v4.1 (http://scrna.sklehabc.com/), rSNPBase (http://rsnp3.psych.ac.cn/) and RegulomeDB (https://www.regulomedb.org/regulome-search/) were used to annotate gene regulatory elements^14–17^. GTEx (https://gtexportal.org/home/) was used to identify expression quantitative trait locus (eQTL) amongst significant variants^51^. Hi-C data of K562 cells were generated from 3D Genome Browser (http://3dgenome.fsm.northwestern.edu/)^52^. Atlas of Human Blood Cells (http://scrna.sklehabc.com/) and HemaExplorer (http://scrna.sklehabc.com/) were used to investigate the expression of *ARID3A* expression in immune cells^18,19^. JASPAR (https://jaspar.genereg.net/), TRANSFAC (https://genexplain.com/transfac/) and CIS-BP database (http://cisbp.ccbr.utoronto.ca/) were used for motif analysis^53–55^. The transcriptome profiling is available in the Gene Expression Omnibus database under accession codes GSE119600. The genotyping data have been deposited in China National Genomics Data Center under accession code OMIX002908 (https://ngdc.cncb.ac.cn/).
