# Supplementary material for: A regulatory variant at 19p13.3 is associated with primary biliary cholangitis risk and ARID3A expression
Source: Nat Commun. 2023 Mar 28;14:1732. doi: 10.1038/s41467-023-37213-5 (PMC10049997; doi:10.1038/s41467-023-37213-5)
Supplement: Supplementary file 1 — Supplementary Information [file 41467_2023_37213_MOESM1_ESM.pdf]

Supplementary Figure

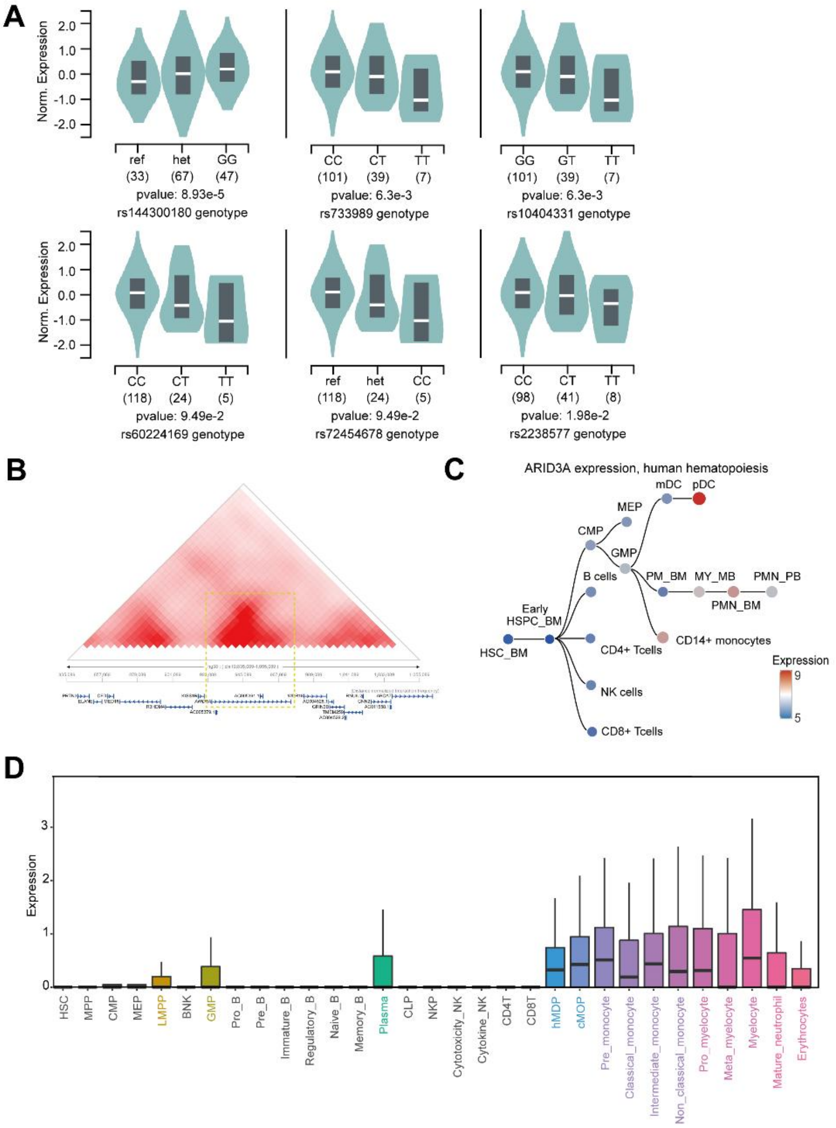

Supplementary Figure 1. Candidate variants at 19p13.3 in addition to rs2238574.

(A) Association between the expression of *ARID3A* and genotypes of rs144300180, rs733989, rs10404331, rs60224169, rs72454678 and rs2238577 in EBV transformed lymphocytes. Source data are derived from GTEx. (B) Three-dimensional chromatin interactions for the *ARID3A* locus in K562 cells. Source data are derived from 3D Genome Browser. (C) *ARID3A* is highly expressed in myeloid cells and markedly up-regulated in differentiated myeloid cells. Source data are derived from HemaExplorer. (D) *ARID3A* is highly expressed in myeloid cells from the single-cell reference of human blood cells. Source data are derived from Atlas of Human Blood

Cells.

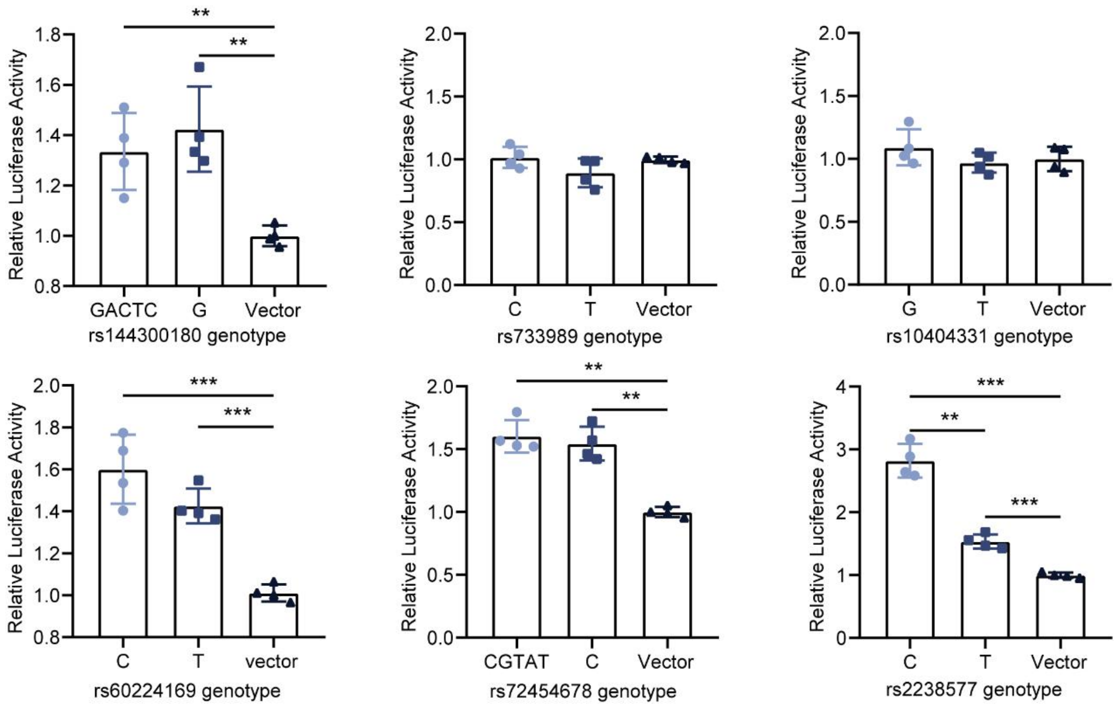

Supplementary Figure 2. Luciferase reporter assay for pGL3 plasmids containing rs144300180, rs733989, rs10404331, rs60224169, rs72454678 and rs2238577. Data are representative of three independent experiments (Mean ± SD for quadruplicates). P values are calculated using unpaired two-tailed Student's t test and indicated significant if $p < 0.05$ (*), $p < 0.01$ (**), or $p < 0.001$ (***).

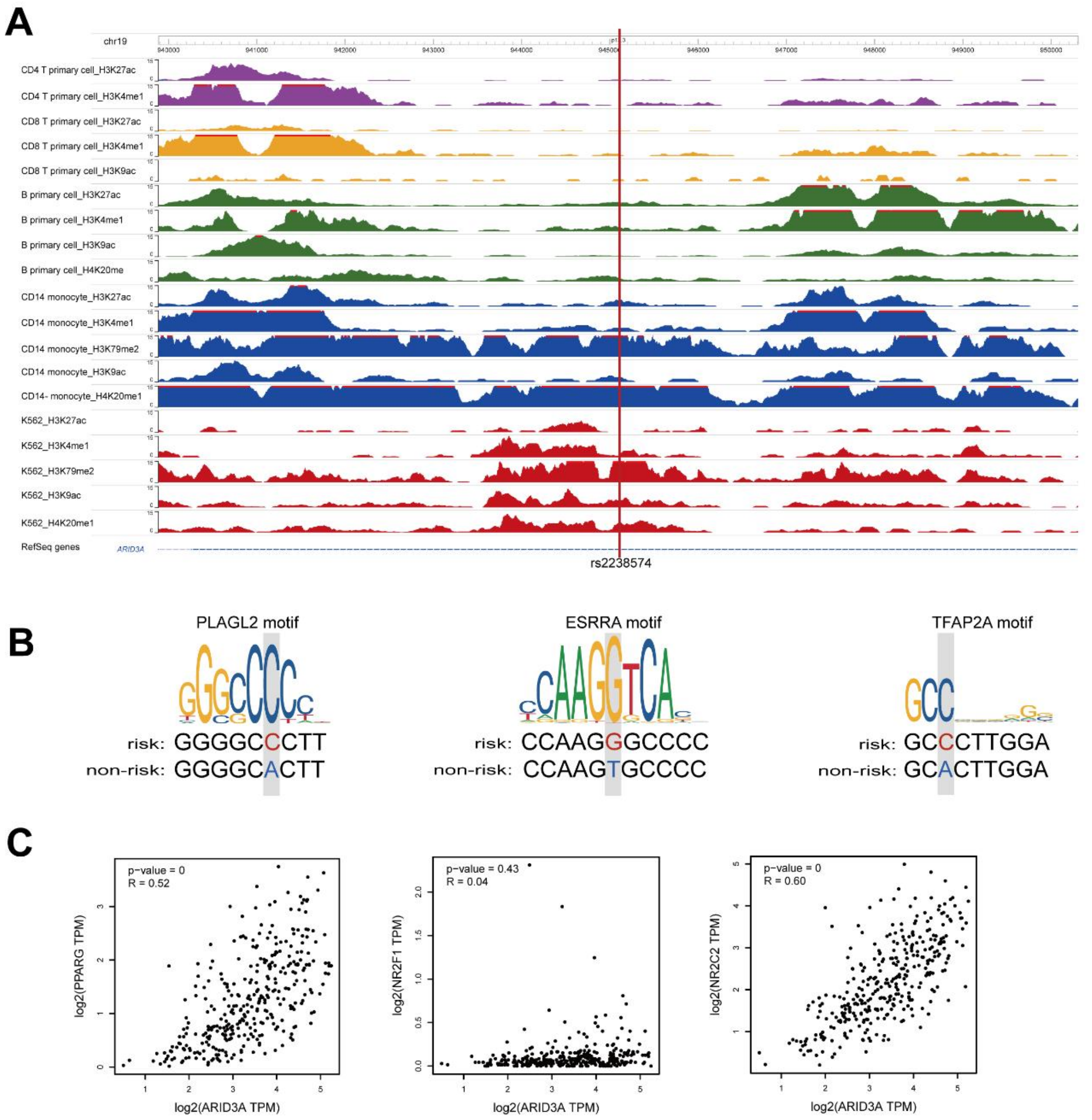

Supplementary Figure 3. The risk allele of rs2238574 exhibits higher binding affinity for transcription factors.

(A) ChIP-seq tracks showing the enrichment of active enhancer marks at the rs2238574-containing region. Source data are derived from ENCODE. (B) Motif analysis suggests that rs2238574 resides within DNA-binding motifs of TFAP2A, PLAGL2 and ESRRA. (C) Scatterplots showing expression correlations between *ARID3A* and *PPARG*, *NR2F1* and *NR2C2* in whole blood. Source data are derived from GEPIA. P values are calculated using Pearson's correlation.

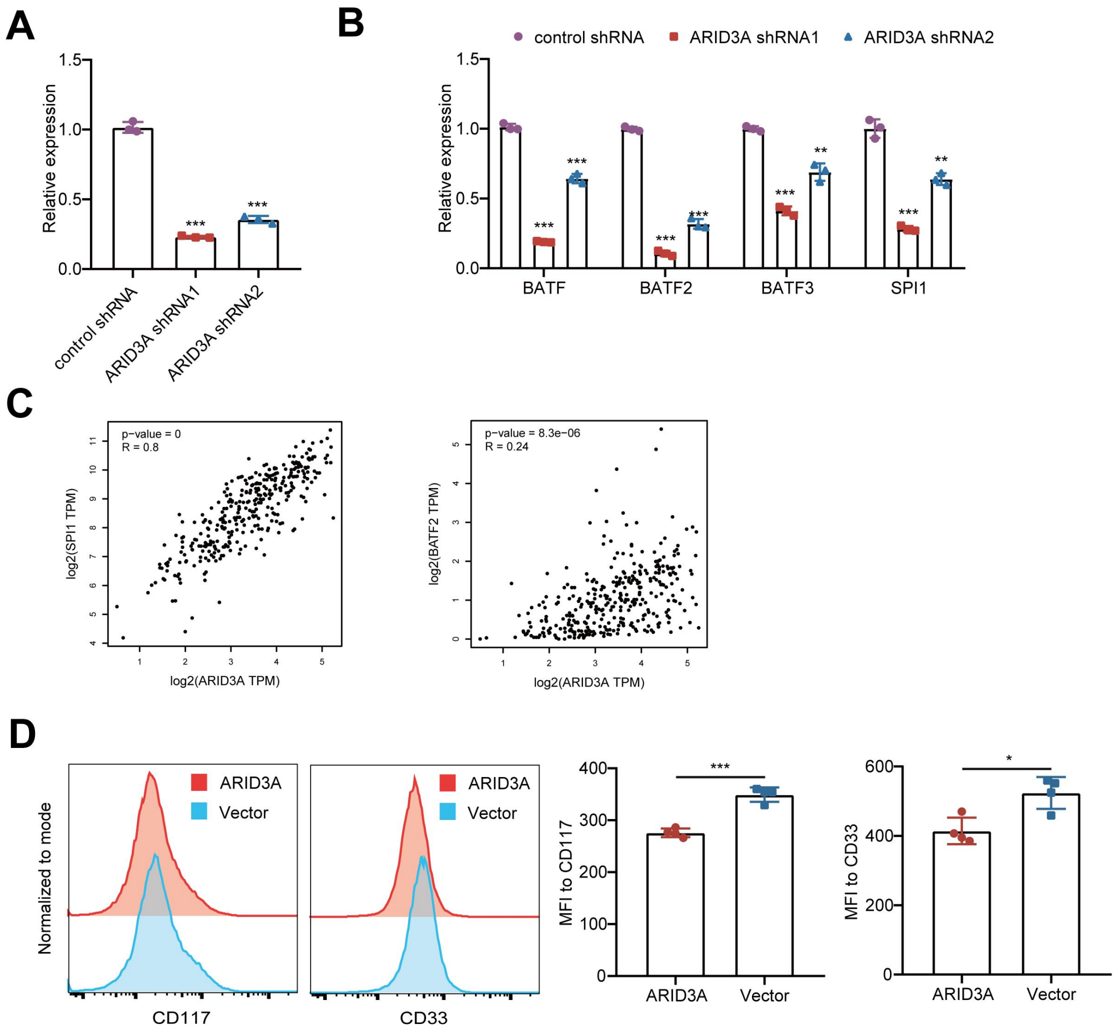

Supplementary Figure 4. ARID3A is associated with differentiation of myeloid cells. (A) Decreased expression of *ARID3A* after *ARID3A* shRNA-mediated knock-down in K562 cells. (B) Relative expression of *BATF*, *BATF2*, *BATF3* and *SPI1* after *ARID3A* shRNA-mediated knock-down in K562 cells. (C) Scatterplots showing expression correlations between *ARID3A* and *BATF2* or *SPI1* in whole blood. Source data are derived from GEPIA. (D) Flow cytometric measurement of surface antigen CD117 and CD33 after *ARID3A* plasmid overexpression in K562 cells. Data are representative of three independent experiments (Mean ± SD for triplicates or quadruplicates). P values are calculated using unpaired two-tailed Student's t test or Pearson's correlation, where applicable. *$p < 0.05$, **$p < 0.01$, ***$p < 0.001$. Abbreviation: MFI, mean fluorescence intensity.

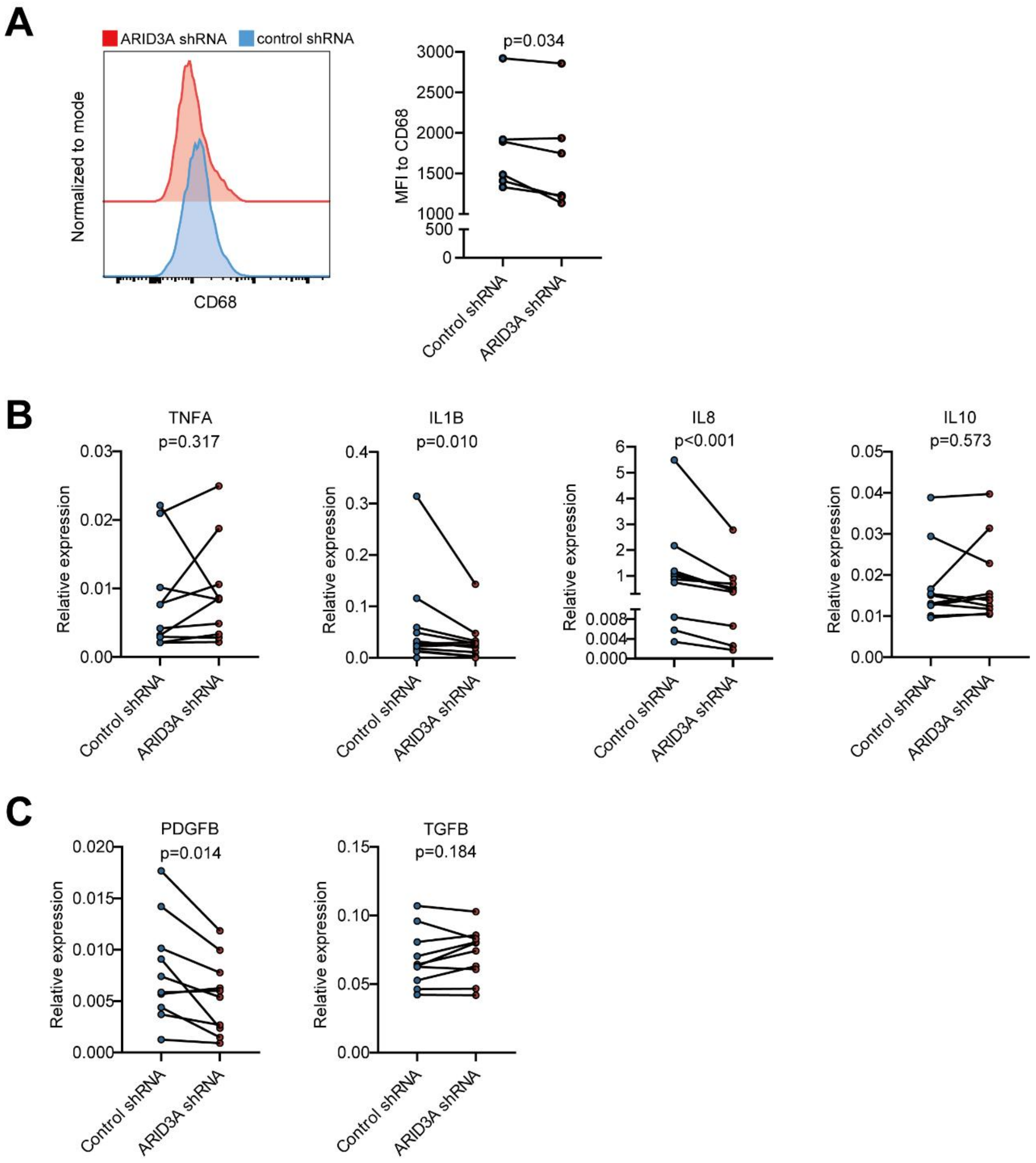

Supplementary Figure 5. ARID3A is associated with differentiation and function of primary myeloid cells.

(A) Flow cytometric measurement of CD68 after knock-down of *ARID3A* in primary human monocytes (n=6). (B) Relative expression of *TNFA*, *IL1B*, *IL8* and *IL10* in *ARID3A* knock-down MDMs (n=10). (C) Relative expression of *PDGFB* and *TGFB* in *ARID3A* knock-down MDMs (n=10). P values are calculated using paired two-tailed Student's t test. Abbreviation: MFI, mean fluorescence intensity. MDMs, monocyte-derived macrophages.

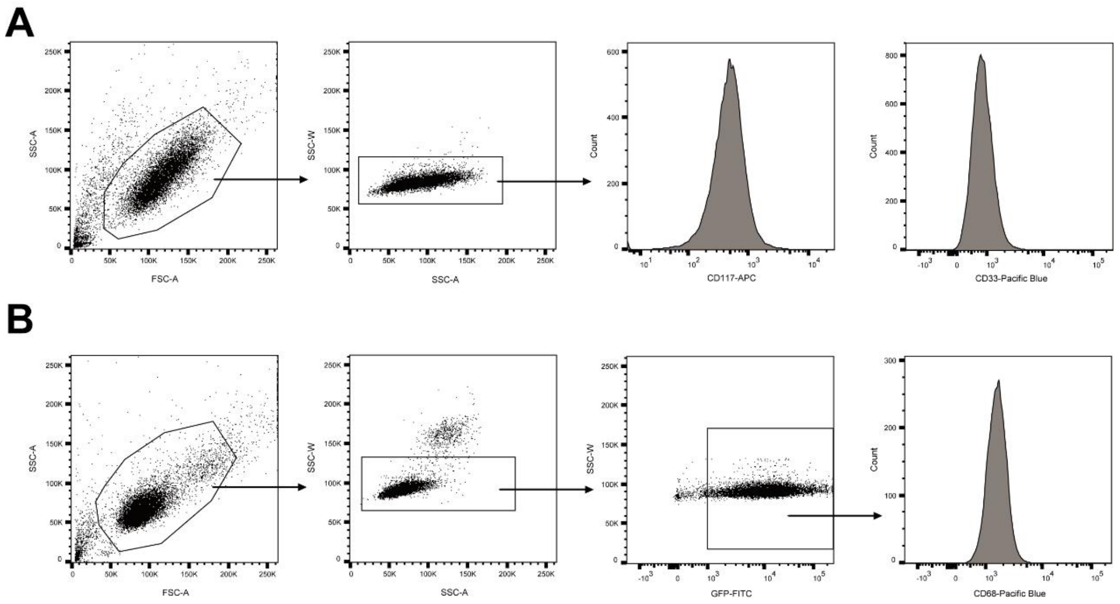

Supplementary Figure 6. Manual gating scheme.

(A) Gating strategy for identification of CD117⁺ cells or CD33⁺ cells in K562 cells. (B) Gating strategy for identification of CD68⁺ cells in primary human monocytes.

Supplementary Table

Supplementary Table 1. SNP at 19p13.3 reaching genome-wide significance.

Chr	SNP	Position (bp)*	A1	A2	Cohort 1				Cohort 2				Meta analysis	
					Case Freq_A1	Control Freq_A1	P-value	OR (95% CI)	Case Freq_A1	Control Freq_A1	P-value	OR (95% CI)	P-value	OR (95% CI)
19	rs144300180	927929	GACTC	G	0.494	0.551	1.56×10^{-8}	0.73 (0.62-0.84)	0.499	0.535	3.76×10^{-2}	0.86 (0.73-1.00)	1.55×10^{-8}	0.73 (0.62-0.84)
19	rs2238571	930874	C	T	0.496	0.554	6.13×10^{-8}	0.75 (0.54-0.85)	0.497	0.550	1.37×10^{-3}	0.81 (0.69-0.94)	5.24×10^{-10}	0.77 (0.69-0.85)
19	rs2238572	931407	G	A	0.538	0.483	1.80×10^{-7}	1.32 (1.22-1.43)	0.548	0.497	4.01×10^{-3}	1.20 (1.07-1.33)	5.01×10^{-9}	1.27 (1.19-1.35)
19	rs3216345	931409	G	GCC	0.538	0.483	1.98×10^{-7}	1.32 (1.22-1.43)	0.547	0.497	3.98×10^{-3}	1.20 (1.08-1.33)	5.45×10^{-9}	1.27 (1.19-1.35)
19	rs350146	931523	C	T	0.481	0.537	4.51×10^{-7}	0.77 (0.67-0.87)	0.481	0.540	1.33×10^{-3}	0.82 (0.70-0.94)	2.51×10^{-9}	0.79 (0.71-0.87)
19	rs5015042	931702	A	G	0.538	0.483	2.30×10^{-7}	1.32 (1.22-1.43)	0.546	0.496	3.22×10^{-3}	1.21 (1.08-1.33)	4.79×10^{-9}	1.27 (1.19-1.35)
19	rs113703584	936297	G	A	0.585	0.521	1.82×10^{-8}	1.36 (1.26-1.47)	0.594	0.550	8.38×10^{-4}	1.36 (1.26-1.47)	1.88×10^{-8}	1.36 (1.26-1.47)
19	rs10404470	937033	T	C	0.593	0.527	1.02×10^{-8}	1.37 (1.26-1.47)	0.611	0.569	9.02×10^{-4}	1.27 (1.13-1.41)	1.00×10^{-8}	1.37 (1.26-1.47)
19	rs733989	938708	C	T	0.589	0.521	1.21×10^{-8}	1.36 (1.25-1.46)	0.603	0.561	7.93×10^{-4}	1.28 (1.13-1.42)	1.20×10^{-8}	1.35 (1.25-1.46)
19	rs10402860	939279	C	T	0.602	0.537	2.64×10^{-8}	1.34 (1.24-1.45)	0.630	0.588	1.95×10^{-3}	1.26 (1.11-1.40)	2.71×10^{-8}	1.34 (1.24-1.45)
19	rs10414193	939697	A	G	0.597	0.532	4.67×10^{-8}	1.33 (1.23-1.44)	0.618	0.577	2.06×10^{-3}	1.25 (1.11-1.40)	4.64×10^{-8}	1.33 (1.23-1.44)
19	rs10404331	940927	G	T	0.584	0.517	2.76×10^{-8}	1.34 (1.24-1.44)	0.602	0.563	1.72×10^{-3}	1.26 (1.12-1.40)	2.81×10^{-8}	1.34 (1.24-1.44)
19	rs10404332	940928	G	C	0.584	0.517	2.76×10^{-8}	1.34 (1.24-1.44)	0.602	0.563	1.72×10^{-3}	1.26 (1.12-1.40)	2.81×10^{-8}	1.34 (1.24-1.44)
19	rs60224169	941423	C	T	0.600	0.536	4.48×10^{-8}	1.33 (1.23-1.44)	0.631	0.593	3.13×10^{-3}	1.25 (1.10-1.39)	4.52×10^{-8}	1.33 (1.23-1.44)
19	rs10415976	941603	A	G	0.587	0.519	2.14×10^{-8}	1.34 (1.24-1.45)	0.610	0.571	1.43×10^{-3}	1.26 (1.12-1.41)	2.08×10^{-8}	1.34 (1.24-1.45)
19	rs72454678	941825	CGTAT	C	0.600	0.535	4.08×10^{-8}	1.34 (1.23-1.44)	0.631	0.592	3.12×10^{-3}	1.25 (1.10-1.39)	4.20×10^{-8}	1.34 (1.23-1.44)
19	rs7258301	942660	A	G	0.585	0.517	1.65×10^{-8}	1.35 (1.25-1.45)	0.586	0.550	2.99×10^{-3}	1.25 (1.11-1.40)	1.69×10^{-8}	1.35 (1.25-1.45)
19	rs2238574	945089	C	A	0.581	0.512	8.36×10^{-9}	1.36 (1.26-1.47)	0.579	0.543	2.46×10^{-3}	1.26 (1.11-1.41)	8.29×10^{-9}	1.36 (1.26-1.47)
19	rs4806860	945710	C	T	0.576	0.508	9.64×10^{-9}	1.36 (1.26-1.47)	0.569	0.533	2.13×10^{-3}	1.27 (1.12-1.42)	9.82×10^{-9}	1.37 (1.25-1.47)
19	rs2238577	948532	C	T	0.564	0.502	4.61×10^{-8}	1.35 (1.24-1.46)	0.581	0.546	3.03×10^{-3}	1.26 (1.11-1.41)	4.63×10^{-8}	1.35 (1.24-1.46)

Chr, chromosome; A1, allele 1; A2, allele 2; OR, odds ratio; CI, confidence interval. * According to GRCh37/hg19 assembly.
