# Supplementary material for: A regulatory variant at 19p13.3 is associated with primary biliary cholangitis risk and ARID3A expression
Source: Nat Commun. 2023 Mar 28;14:1732. doi: 10.1038/s41467-023-37213-5 (PMC10049997; doi:10.1038/s41467-023-37213-5)
Supplement: Supplementary file 2 — Reporting Summary [file 41467_2023_37213_MOESM2_ESM.pdf]

nature portfolio

Corresponding author(s): Xiong Ma

Last updated by author(s): Nov 10, 2022

Reporting Summary

Nature Portfolio wishes to improve the reproducibility of the work that we publish. This form provides structure for consistency and transparency in reporting. For further information on Nature Portfolio policies, see our Editorial Policies and the Editorial Policy Checklist.

Statistics

For all statistical analyses, confirm that the following items are present in the figure legend, table legend, main text, or Methods section.

n/a	Confirmed	
☐	☒	The exact sample size (*n*) for each experimental group/condition, given as a discrete number and unit of measurement
☐	☒	A statement on whether measurements were taken from distinct samples or whether the same sample was measured repeatedly
☐	☒	The statistical test(s) used AND whether they are one- or two-sided<br>*Only common tests should be described solely by name; describe more complex techniques in the Methods section.*
☐	☒	A description of all covariates tested
☐	☒	A description of any assumptions or corrections, such as tests of normality and adjustment for multiple comparisons
☐	☒	A full description of the statistical parameters including central tendency (e.g. means) or other basic estimates (e.g. regression coefficient) AND variation (e.g. standard deviation) or associated estimates of uncertainty (e.g. confidence intervals)
☐	☒	For null hypothesis testing, the test statistic (e.g. *F*, *t*, *r*) with confidence intervals, effect sizes, degrees of freedom and *P* value noted<br>*Give P values as exact values whenever suitable.*
☒	☐	For Bayesian analysis, information on the choice of priors and Markov chain Monte Carlo settings
☒	☐	For hierarchical and complex designs, identification of the appropriate level for tests and full reporting of outcomes
☐	☒	Estimates of effect sizes (e.g. Cohen's *d*, Pearson's *r*), indicating how they were calculated

Our web collection on statistics for biologists contains articles on many of the points above.

Software and code

Policy information about availability of computer code

Data collection	Applied Biosystem7900 quantitative PCR system (Applied Biosystems, USA), Illumina Novaseq platform, laser confocal microscopy (Carl Zeiss, Germany), flow cytometry (BD Biosciences, USA)
Data analysis	EIGENSTRAT software, IMPUTE2, the Ricopili pipeline, META v1.761, LocusZoom v1.2, GSEA v4.2.3, FlowJo software v10.6.2 (Tree Star, USA), SPSS 22.0 (SPSS Inc, USA) , RStudio v1.1.463 with R v3.6.3

For manuscripts utilizing custom algorithms or software that are central to the research but not yet described in published literature, software must be made available to editors and reviewers. We strongly encourage code deposition in a community repository (e.g. GitHub). See the Nature Portfolio guidelines for submitting code & software for further information.

Data

Policy information about availability of data

All manuscripts must include a data availability statement. This statement should provide the following information, where applicable:

- Accession codes, unique identifiers, or web links for publicly available datasets
- A description of any restrictions on data availability
- For clinical datasets or third party data, please ensure that the statement adheres to our policy

Encyclopedia of DNA Elements (ENCODE) database, HaploReg v4.1, rSNPBase and RegulomeDB were used to annotate gene regulatory elements14-17. GTEx (release v8) was used to identify expression quantitative trait locus (eQTL) amongst significant variants51. Hi-C and 4C data of K562 cells were generated from 3D Genome Broswer52. Atlas of Human Blood Cells and HemaExplorer were used to investigate the expression of ARID3A expression in immune cells18,19. JASPAR, TRANSFAC and CIS-BP database were used for motif analysis53-55. The remaining sequencing data are available from the corresponding author upon reasonable request.

Field-specific reporting

Please select the one below that is the best fit for your research. If you are not sure, read the appropriate sections before making your selection.

☒ Life sciences ☐ Behavioural & social sciences ☐ Ecological, evolutionary & environmental sciences

For a reference copy of the document with all sections, see nature.com/documents/nr-reporting-summary-flat.pdf

Life sciences study design

All studies must disclose on these points even when the disclosure is negative.

Sample size	Genetic Association Study Power Calculator (https://csg.sph.umich.edu/abecasis/gas_power_calculator/) was used to determine the necessary sample size for clinical sample analysis.
Data exclusions	We performed systematic quality control on the raw genotyping data to filter out both unqualified samples and SNPs using the Ricopili pipeline for case-control groups. Samples with low SNP call rate (<98%) as well as individuals closely related based on estimated identity-by-descent (PI_HAT>0.25) were excluded for further analysis. Samples with inconsistent sex (compared with the sample record) were removed. SNPs with call rates <98%, MAF <0.5%, or significant deviation from Hardy–Weinberg equilibrium (HWE) in cases (p<1×10–10) or controls (p<1×10–6) were excluded.
Replication	All experiments for this study are from multiple biologically independent experiments and biologically independent samples, with "n" and replication numbers reported in the legend of each Figure.
Randomization	Randomization was not relevant to this study as all experiments were performed with molecular/cell biology techniques in cell lines where the experimenter designs and performs the experimental conditions so randomization between conditions is not possible.
Blinding	Investigations were not blinded to group allocation as data analysis involved in all cases objective measurement methods are not affected by investigator bias.

Reporting for specific materials, systems and methods

We require information from authors about some types of materials, experimental systems and methods used in many studies. Here, indicate whether each material, system or method listed is relevant to your study. If you are not sure if a list item applies to your research, read the appropriate section before selecting a response.

Materials & experimental systems

n/a	Involved in the study	
☐	☒	Antibodies
☐	☒	Eukaryotic cell lines
☒	☐	Palaeontology and archaeology
☒	☐	Animals and other organisms
☐	☒	Human research participants
☒	☐	Clinical data
☒	☐	Dual use research of concern

Methods

n/a	Involved in the study	
☒	☐	ChIP-seq
☐	☒	Flow cytometry
☒	☐	MRI-based neuroimaging

Antibodies

Antibodies used	anti-CD68-BV421 (BD Biosciences, #564943, 1:100), anti-CD33-BV421 (BioLegend, #366622, 1:100), anti-CD117-APC (BioLegend, #313232, 1:100), anti-ARID3A (LSBio, #LS-B5399, 5 ug/ml), anti-CD33 (Abcam, #ab269456, 1:200), anti-CD11b (Abcam, #ab133357, 1:400), Mono-Methyl-Histone H3 (Lys4) (D1A9) XP® Rabbit mAb (CST, #5326, 5μg per test), Di-Methyl-Histone H3 (Lys79) (D15E8) XP® Rabbit mAb (CST, #5427, 5μg per test), Histone H3K20me1 (mono-methyl Lys20) antibody (GeneTex, #GTX55480, 5μg per test), Acetyl-Histone H3 (Lys9) (C5B11) Rabbit mAb (CST, #9649, 5μg per test), PPAR Gamma Polyclonal Antibody (Proteintech, #16643-1-AP, 5μg per test), COUP TF1 Antibody (GeneTex, #GTX114835, 5μg per test), Anti-TR4 Antibody (Abcam, #ab109301, 5μg per test), Anti-Transcription factor AP-2-alpha Antibody (Abcam, #ab52222, 5μg per test), PLAGL2 Polyclonal Antibody (Proteintech, #11540-1-AP, 5μg per test), ERRA (E1G1J) Rabbit mAb (CST, #13826, 5μg per test), Normal rabbit IgG (CST, #2729, 5μg per test).
Validation	anti-CD68-BV421 (BD Biosciences, #564943): Application: FC. Species Reactivity: Human.<br>anti-CD33-BV421 (BioLegend, #366622): Application: FC. Species Reactivity: Human.<br>anti-CD117-APC (BioLegend, #313232): Application: FC. Species Reactivity: Human.<br>anti-ARID3A (LSBio, #LS-B5399): Application: IHC, IHC-P, IF, WB, ELISA. Species Reactivity: Human.<br>anti-CD33 (Abcam, #ab269456): Application: IHC-P, ICC/IF, IP, WB. Species Reactivity: Human.<br>anti-CD11b (Abcam, #ab133357): Application: WB, IHC-P. Species Reactivity: Mouse, Rat, Human.<br>Mono-Methyl-Histone H3 (Lys4) (D1A9) XP® Rabbit mAb (CST, #5326): Application: WB, IF, F, CHIP. Species Reactivity: Mouse, Rat, Human.

Di-Methyl-Histone H3 (Lys79) (D15E8) XP® Rabbit mAb (CST, #5427): Application: WB, CHIP. Species Reactivity: Mouse, Rat, Human.
Histone H3K20me1 (mono-methyl Lys20) antibody (GeneTex, #GTX55480): Application: WB, IP, IHC, CHIP, ICC-IF. Species Reactivity: Mouse, Rat, Human.
Acetyl-Histone H3 (Lys9) (C5B11) Rabbit mAb (CST, #9649): Application: WB, IP, IHC, IF, F, CHIP. Species Reactivity: Mouse, Rat, Human.
PPAR Gamma Polyclonal Antibody (Proteintech, #16643-1-AP): Application: CHIP, CoIP, IF, IHC, IP, WB. Species Reactivity: Mouse, Rat, Human.
COUP TF1 Antibody (GeneTex, #GTX114835): Application: WB, IP, CHIP. Species Reactivity: Human, Mouse.
Anti-TR4 Antibody (Abcam, #ab109301): Application: WB, IP, CHIP. Species Reactivity: Mouse, Rat, Human.
Anti-Transcription factor AP-2-alpha Antibody (Abcam, #ab52222): Application: ELISA, ChIP, EMSA, WB, IHC-P, ICC/IF. Species Reactivity: Mouse, Human.
PLAGL2 Polyclonal Antibody (Proteintech, #11540-1-AP): Application: IF, IHC, WB, ELISA, CHIP. Species Reactivity: Mouse, Rat, Human.
ERRA (E1G1J) Rabbit mAb (CST, #13826): Application: WB, CHIP. Species Reactivity: Mouse, Rat, Human.
ESRRA (CST, #13826): Application: WB, IP, IHC, IF, F, CHIP. Species Reactivity: Mouse, Rat, Human.
Normal rabbit IgG (CST, #2729):Application: IP, CHIP. Species Reactivity: Mouse, Rat, Human.

Eukaryotic cell lines

Policy information about cell lines

Cell line source(s)	HEK293T cells and K562 cells, purchased from the Cell Bank of the Chinese Academy of Sciences in Shanghai.
Authentication	All the cell lines were authenticated via short tandem repeat fingerprinting.
Mycoplasma contamination	All the cell lines were tested negative for mycoplasma.
Commonly misidentified lines (See ICLAC register)	No commonly misidentified lines were used in this study.

Human research participants

Policy information about studies involving human research participants

Population characteristics	For meta-analysis:<br>1931 PBC subjects (87.4% female, the age range from 22-85) and 7852 controls (53.2% female, the age range from 15-96) in two Han Chinese cohorts. Both cases and controls in cohort 1 were genotyped with Han Chinese population-specific HumanOmniZhongHua-8 BeadChip v1.1, and cohort 2 were genotyped using Infinium Global Screening Array v3.0. Age, sex and ten principal components were used as covariates in the association analysis to correct for the population stratification.<br>For histological staining:<br>89 patients with PBC, 42 with AIH, 20 with CHB, and 10 HC The disease history (range from 0-151 month for PBC) was used as a covariate in the association analysis.
Recruitment	Cases were recruited from Renji Hospital and controls were recruited from Shanghai Jiao Tong University and matched to cases by age and gender. All participants were genetically unrelated individuals of self-claimed Chinese Han descent.
Ethics oversight	The study was approved by the research ethics boards of Renji Hospital, Shanghai Jiao Tong University.

Note that full information on the approval of the study protocol must also be provided in the manuscript.

Flow Cytometry

Plots

Confirm that:

☒ The axis labels state the marker and fluorochrome used (e.g. CD4-FITC).

☒ The axis scales are clearly visible. Include numbers along axes only for bottom left plot of group (a 'group' is an analysis of identical markers).

☒ All plots are contour plots with outliers or pseudocolor plots.

☒ A numerical value for number of cells or percentage (with statistics) is provided.

Methodology

Sample preparation	Cells were harvested and washed with PBS before staining and then incubated with antibodies for 30 min in the dark at 4 °C. For intracellular staining, surface-stained cells were fixed and permeabilized with Cytofix/Cytoperm solution (BD Biosciences, USA) for 20 min at 4°C and then were stained with antibodies for 30 min at 4°C. Subsequently, cells were finally washed with PBS and then analyzed by flow cytometry.
Instrument	flow cytometry (BD Biosciences, USA)
Software	FlowJo software v10.6.2 (Tree Star, USA).

Cell population abundance	A total of 500,000 events were recorded and analyzed. Considering that there were no obvious cell populations for CD117+ cells , CD33+ cells or CD68+ cells, we used Mean Fluorescent Intensity (MFI) to measure the shift in fluorescence intensity of the population of cells.
Gating strategy	Cells were initially gated based on forward and side scatter profiles. Positive and negative cell populations were gated based on staining the fluorescently-conjugated monoclonal antibodies.

☒ Tick this box to confirm that a figure exemplifying the gating strategy is provided in the Supplementary Information.
